# New Generation Antibiotics Derived from DABCO-Based Cationic Polymers

**DOI:** 10.3390/antibiotics14090856

**Published:** 2025-08-25

**Authors:** Betul Zehra Temur, Ilay Ceren Cetinkaya, Merve Acikel Elmas, Nihan Unubol, Serap Arbak, Tanil Kocagoz, Tarik Eren, Ozge Can

**Affiliations:** 1 Department of Medical Biotechnology, Institute of Health Sciences, Acibadem Mehmet Ali Aydinlar University, Istanbul 34638, Türkiye; betulzkarakus@gmail.com (B.Z.T.); tanilkocagoz@gmail.com (T.K.); 2 Department of Chemistry, Faculty of Science and Arts, Yildiz Technical University, Istanbul 34220, Türkiye; ilaycerenacar@gmail.com; 3 Department of Histology and Embryology, School of Medicine, Acibadem Mehmet Ali Aydinlar University, Istanbul 34638, Türkiye; merve.elmas@acibadem.edu.tr (M.A.E.); serap.arbak@acibadem.edu.tr (S.A.); 4 Medical Laboratory Techniques, Vocational School of Health Services, Acibadem Mehmet Ali Aydinlar University, Istanbul 34638, Türkiye; nihan.unubol@acibadem.edu.tr; 5 Department of Medical Microbiology, School of Medicine, Acibadem Mehmet Ali Aydinlar University, Istanbul 34638, Türkiye; 6 Department of Biomedical Engineering, Faculty of Engineering and Natural Sciences, Acibadem Mehmet Ali Aydinlar University, Istanbul 34638, Türkiye

**Keywords:** antimicrobial polymer, ROMP, structure-property relationship, cationic polymers, DABCO

## Abstract

**Background/Objectives**: The growing threat of antibiotic resistance necessitates the development of novel antimicrobial agents that effectively target pathogenic microorganisms while minimizing toxicity. **Methods**: Two series DABCO-based cationic homopolymers (D-subs 1kDa, D-subs 5kDa, D-subs 15kDa) and DABCO–pyridinium-based copolymers (PyH-subs 5kDa_Dsubs 5kDa, PyH-subs 7kDa_Dsubs 3kDa, PyH-subs 3kDa_Dsubs 7kDa) were synthesized to mimic to host-defense cationic peptides via ring-opening metathesis polymerization (ROMP). The antimicrobial activities of these polymers were determined by their minimum inhibitory concentrations (MICs) against *E. coli* (Gram-negative bacteria), *P. aeruginosa* (Gram-negative bacteria), *S. aureus* (Gram-positive bacteria), and *C. albicans* (fungus). In vitro cytotoxicity assays revealed selective toxicity towards bacterial cells, with high selectivity indices for several copolymers. To gain insight into the mechanism of action, morphological changes in *S. aureus* upon exposure to D-subs 1kDa were examined using scanning electron microscopy (SEM) and transmission electron microscopy (TEM). **Results**: The D-subs 15kDa homopolymer demonstrated the highest overall antimicrobial activity, particularly against *S. aureus* (MIC: 8 µg/mL), with all polymers exhibiting minimal hemolytic activity (HC_50_ ≥ 1024 µg/mL). SEM and TEM results revealed membrane disruption indicative of polymer–bacteria interactions. Additionally, stability studies confirmed polymer integrity under physiological conditions for at least 28 days. **Conclusions**: These results support the potential of DABCO-based cationic polymers as a promising platform for next-generation antimicrobial therapeutics.

## 1. Introduction

Infectious diseases have re-emerged as an important health problem after a century-long decline [1]. As a result of the irresponsible and excessive use of antibiotics, bacterial pathogens have rapidly developed antibiotic resistance [2,3]. According to surveillance data from the European Centre for Disease Prevention and Control (ECDC), approximately 7% of patients admitted to acute care hospitals in Europe develop healthcare-associated infections (HAIs), with national rates ranging between 3% and 10% across member states [4]. This corresponds to an estimated 4.5 million HAI cases annually within the EU, leading to a substantial health burden, including approximately 37,000 direct deaths and an additional 110,000 deaths indirectly linked to HAIs each year. Furthermore, healthcare-associated infections constitute a substantial global burden, with surgical site infections, bloodstream infections, and ventilator-associated pneumonia being among the most prevalent types. These infections are frequently underestimated in routine surveillance systems and necessitate the implementation of structured reporting mechanisms at the national level to inform effective policy and control strategies [5].

Next-generation synthetic antibiotics are needed to overcome bacterial resistance and prevent the further spread of infections [6,7,8]. These advanced agents are designed to prevent the development of bacterial resistance and restrict the spread of infections to wider microbial populations. Nowadays, there are two major research topics in the field of antimicrobial chemical therapies, apart from traditional antibiotics based on small molecules. The first strategy involves the synthesis of natural host defense peptides (HDPs) and their derivatives. Natural antimicrobial peptides exhibit a characteristic balance of hydrophobic and hydrophilic (positively charged) regions, which is critical for their function [9,10,11,12,13].

The specific spatial arrangement and proportion of these moieties play a key role in maximizing antimicrobial efficacy while minimizing cytotoxicity. Importantly, HDPs have demonstrated broad-spectrum efficacy and a reduced tendency to induce resistance due to their non-specific mechanism of action, often involving membrane destabilization rather than targeted biochemical pathways. However, their clinical translation remains limited by issues such as high production costs, enzymatic degradation, and potential cytotoxicity. To overcome these limitations, a second strategy has emerged: the design of synthetic cationic polymers that structurally and functionally mimic HDPs. These polymers aim to replicate the amphiphilic architecture of HDPs, incorporating both cationic moieties to promote electrostatic interactions with bacterial membranes and hydrophobic domains to enable membrane insertion and disruption [14,15,16,17,18].

One widely accepted mechanism through which these polymers exert their bactericidal effect is the “carpet mechanism” where polymers align on the bacterial membrane surface and induce membrane disintegration by forming pores or defects, ultimately leading to cell lysis [19,20,21,22,23]. While other mechanisms also exist, this physical mode of action significantly reduces the likelihood of resistance development. Although the development of resistance mechanisms has been observed in some cases, it is generally believed that bacteria are unlikely—or find it difficult—to develop resistance against amphiphilic polymers due to their non-specific, membrane-disruptive mode of action [14,24].

In the literature, there are various synthetic antimicrobial peptide mimics with high activity and selectivity, synthesized through different polymerization techniques [14,23,24,25,26]. One such method is ring-opening metathesis polymerization (ROMP), a controlled polymerization technique that enables the synthesis of amphiphilic polymers with defined molecular weights, low dispersity (i.e., narrow chain-length distribution), and a well-balanced composition of hydrophilic and hydrophobic groups [27,28,29,30,31,32,33,34].

Previously, our group synthesized cationic homopolymers and copolymers via ROMP, using monomers bearing pyridine-based mono-charged and DABCO-based di-charged quaternary ammonium groups [35]. Notably, the DABCO-containing polymers (MWs: 3000 and 10,000 g/mole), which carry double cationic charges, exhibited the highest antimicrobial activity against *S. aureus* (MIC = 8 μg/mL) and demonstrated high selectivity (selectivity index > 250). The non-toxic nature of these polymers was further supported by hemolytic activity assays (HC_50_ ≥ 1000 μg/mL) and MTT cytotoxicity tests.

In this study, we focused on the antimicrobial activity of DABCO-based cationic homopolymers with varying molecular weights, as well as their copolymers with pyridine-derived hexyl side chains, aiming to enhance antimicrobial efficacy and selectivity against *E. coli*. Scanning electron microscopy (SEM) and transmission electron microscopy (TEM) were used to investigate bacterial morphological changes following polymer treatment. Additionally, the stability of these polymers under physiological conditions was evaluated.

## 2. Results

**Figure 1:**
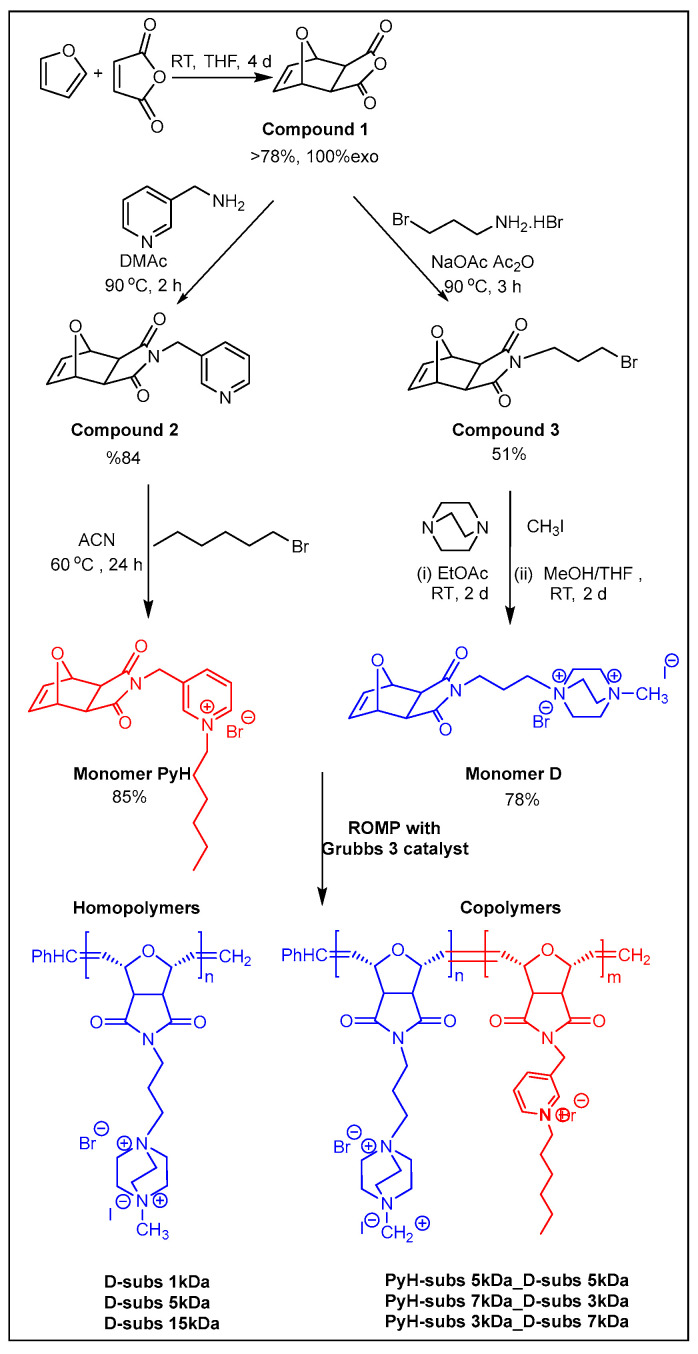
Synthetic pathway for DABCO-based homopolymers and DABCO–PyH copolymers.

Figure 1 illustrates the synthetic pathway of the DABCO-based monomer and its corresponding homopolymers, as well as the copolymerization process involving the hexyl-substituted quaternary pyridine monomer (PyH). The DABCO-based monomer and homopolymers were previously synthesized by our group [35]. In this study, DABCO homopolymers were synthesized with targeted molecular weights of 1000, 5000, and 15,000 g/mol. Similarly, the hexyl pyridinium-based monomer and its copolymers were synthesized for their antimicrobial activities [34].

### 2.1. Characterization of Polymers

Monomer PyH and Monomer D were synthesized and characterized according to the previously reported procedures [34,35,36].

**Figure 2:**
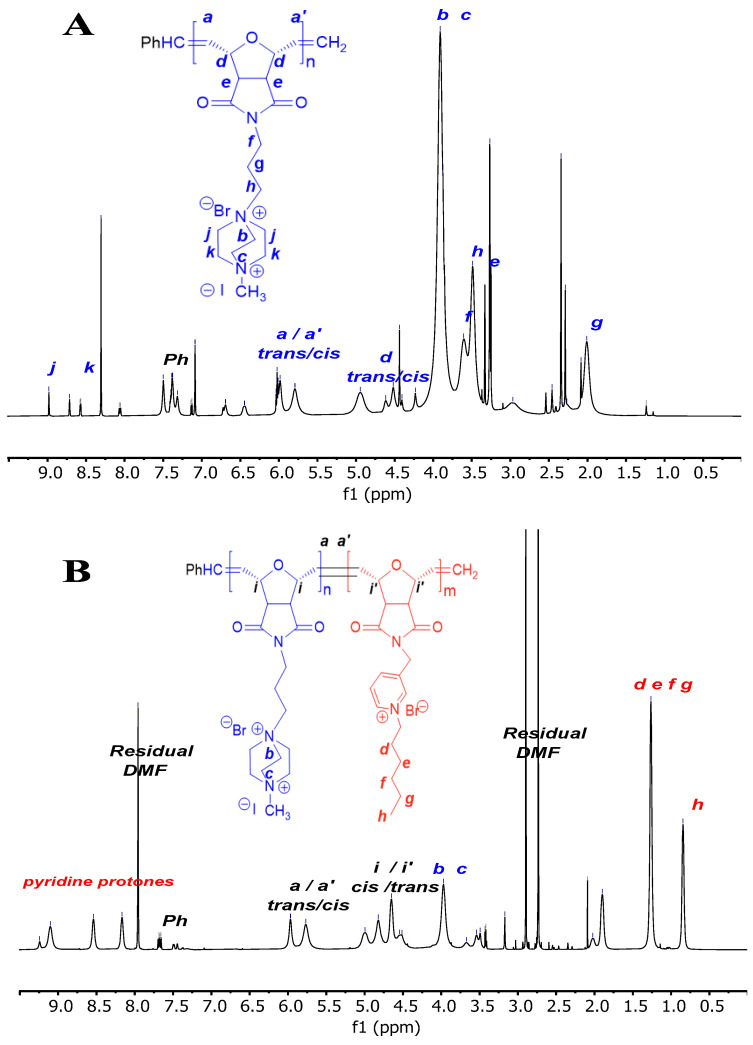
(**A**) ^1^H NMR spectrum of the structure of D-subs 1kDa and (**B**) PyH-subs 7kDa_D-subs 3kDa.

Characterization of the polymers was carried out by ^1^H NMR techniques. Representative ^1^H NMR assignments are shown in Figure 2. The disappearance of the olefin peaks (–CH=CH–) of monomer D were observed at the ^1^H NMR spectrum at 6.5 and 5.1 ppm, and the appearance of the polymer backbone double bond protons stereoisomers (cis and trans) was observed for homopolymers (D-subs 1kDa, D-subs 5kDa and D-subs 15kDa) in the ranges from 5.8 to 4.5 ppm and from 6.1 to 4.9 ppm. Characteristic end-group phenyl protons of the homopolymers were observed in a range from 7.3 to 7.5 ppm.

In the copolymers, the disappearance of the olefinic protons of the norbornene ring at 6.5 ppm and the appearance of new peaks at approximately 5.6 ppm (cis) and 6.0 ppm (trans) confirmed the occurrence of ring-opening polymerization.

The molecular weight of the polymers was calculated using NMR techniques. M_n_ values were calculated via end-group analysis from the ^1^H NMR spectra of each polymer (Table 1).

The molecular weight of homopolymers was calculated by multiplying the ratio of integrals per proton in repeating units (DP) with the molecular weight of these groups (Equations (1) and (2)).

(1) $$M_{n}=DP\times {MW}_{monomer}$$

(2) $$DP=\frac{\left[\left(Integral\;of\;the\;olefinic\;region/2\;protons\right)\right]}{\left[(Integral\;of\;styrene\;end\;group\;region/5\;protons\right]}$$

The phenyl and olefinic groups in the Grubbs catalyst form the end group of polymers. Phenyl group protons (5H) were observed around 7.5 ppm. In addition, olefinic protons were seen at around 4.5–5.8 ppm corresponding to two protons. The styrene end group divided by five gave the value of the integrals per proton. We determined the molecular weight of the D-subs 1kDa, D-subs 5kDa and D-subs 15kDa as 1239 g mol^−1^, 3304 g mol^−1^, and 7434 g mol^−1^, respectively.

Using these methods, the M_n_ values of the D-subs 1kDa, D-subs 5kDa, and D-subs 15kDa homopolymers were determined as 1239 g/mol, 3304 g/mol, and 7434 g/mol, respectively. Copolymer composition was evaluated by integrating monomer-specific signals in the ^1^H-NMR spectrum. For example, hexyl pyridinium CH_3_ and CH_2_ protons appeared at 0.88 ppm and 1.25 ppm, respectively, while the DABCO-based polymer’s characteristic N–CH_2_ protons appeared at approximately 4.3–4.1 ppm.

The M_n_ values for PyH-subs 5kDa_D-subs 5kDa, PyH-subs 7kDa_D-subs 3kDa, and PyH-subs 3kDa_D-subs 7kDa copolymers were determined as 6574 g/mol, 8548 g/mol, and 13,167 g/mol, respectively.

**Figure 3:**
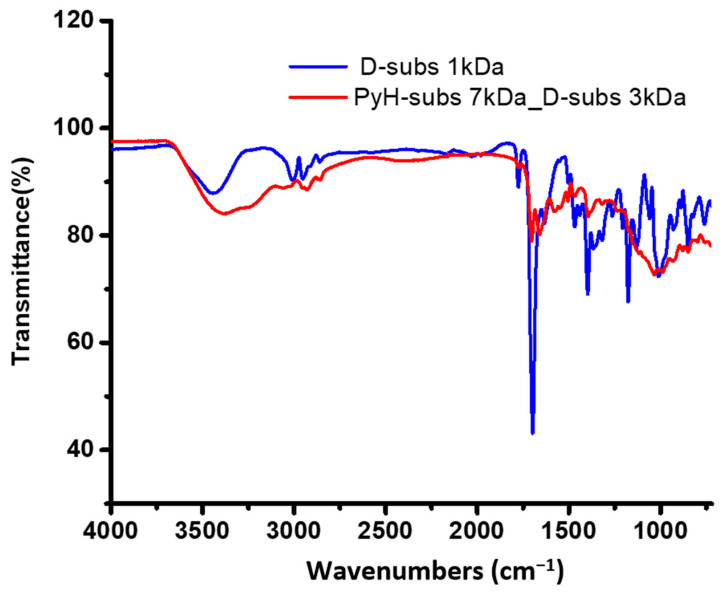
FTIR spectrums of the structure of D-subs 1kDa and PyH-subs 7kDa_D-subs 3kDa.

A structural analysis of the copolymers was also carried out using FTIR spectroscopy (Figure 3). Both D-subs 1kDa and PyH-subs 7kDa_Dsubs 3kDa showed characteristic peaks corresponding to the quaternary ammonium N-H group’s absorption band, centered around 3435 cm^−1^, were observed. These bands typically appear in the 3400–3450 cm^−1^ region, which is in line with earlier studies on quaternized ROMP polymers [30]. In addition to this, consistent asymmetric and symmetric CH_2_ stretching vibrations of aliphatic chains were observed at 2894 cm^−1^ and 2825 cm^−1^, respectively. C=O stretching bands related to imide (N–C=O–R) functionalities at 1776 cm^−1^, 1698 cm^−1^, and 1634 cm^−1^ indicated the presence of imide-containing oxanorbornene monomers [28].

As a characteristic feature, the C-N-C stretching vibrations from the DABCO unit detected at 1058 cm^−1^ is weaker in the PyH-subs copolymer. Similar peaks between 1050 and 1100 cm^−1^ were shown for other quaternized DABCO derivatives [37]. Additional peaks at 1318.1 cm^−1^ and 1366.0 cm^−1^ correspond to C–N–C stretching within the imide ring and were observed in both polymers [38].

Upon incorporation of the hexyl pyridine unit, an increase in the intensity of aliphatic peaks associated with the alkyl chain was noted in the copolymer structure. Additionally, the characteristic C-H stretching vibrations of the phenyl ring were observed around 720 cm^−1^ [37].

The particle size values measured by dynamic light scattering (DLS) using a zeta-sizer provide important insights into the self-assembly behavior and solution properties of the synthesized polymers. As shown in Table 1, the hydrodynamic diameters (d.nm) increased with increasing molecular weight for the homopolymers. This trend suggests that higher molecular weight DABCO-based homopolymers tend to form larger aggregates or self-assembled structures in solutions, likely due to increased chain length and enhanced inter- and intramolecular interactions (e.g., electrostatic and hydrophobic effects). For the copolymers containing hexyl pyridinium and DABCO cationic units, the particle sizes vary more significantly depending on the hydrophilic/hydrophobic balance (as reflected by the *m*/*n* feed ratio) and the total molecular weight. Interestingly, Pyrsubs7kDa_Dsubs3kDa, despite having a relatively high M_n_ (8548 g/mol), shows a much smaller particle size (173.5 nm) compared with the others. This suggests a more uniform and possibly micelle-like self-assembly structure, where a better-defined amphiphilic balance (with higher hydrophobic content) could result in compact nanoparticles with reduced aggregation. In contrast, the other copolymers exhibit larger particle sizes, which may result from aggregates or irregular structures.

These observations indicate that both the total molecular weight and the *m*/*n* ratio (hydrophobic/hydrophilic balance) are critical in dictating polymer aggregation behavior in aqueous media.

### 2.2. Removal of Residual Ruthenium (Ru) from Polymers

To address the issue of residual Ru in ROMP-based polymers, we applied two purification techniques: dialysis membranes and activated carbon treatment (Table 2) [39]. DABCO-based homopolymers were selected for this study due to their favorable hemolytic concentration values. According to ICP-MS analysis, both purification methods effectively reduced the Ru content, with activated carbon treatment proving more efficient than dialysis. Additionally, a correlation was observed between molecular weight and the degree of Ru removal; the high molecular weight polymer D-subs 15kDa showed superior performance in both methods.

### 2.3. Antimicrobial Activity of Polymers

The antimicrobial activity of synthesized polymers was tested against *E. coli* ATCC 25922, *S. aureus* ATCC 29213, *P. aeruginosa* ATCC 27853, and *C. albicans* ATCC 10231. The MIC values varied according to the type of polymer and the microorganism tested and are summarized in Table 3.

DABCO homopolymers (D-subs 1kDa, D-subs 5kDa, D-subs 15kDa) exhibited antimicrobial activity against *S. aureus*, with MIC values of 8 or 16 µg/mL. The absence of an outer membrane in *S. aureus* makes it more susceptible to direct interaction with cationic polymers. D-subs 15kDa showed the highest potency, particularly against *E. coli* (MIC: 16 µg/mL) and *P. aeruginosa* (MIC: 64 µg/mL), likely due to its higher charge density and increased multivalent interactions, which enhance membrane disruption efficiency.

In the case of the DABCO–pyridinium copolymers, the antimicrobial performance varied depending on the DABCO-to-pyridinium ratio. The most effective copolymer, PyH-subs 7kDa_Dsubs 3kDa, exhibited strong activity against all tested strains (*E. coli*: 32 µg/mL, *P. aeruginosa*: 32 µg/mL, *S. aureus*: 16 µg/mL, *C. albicans*: 256 µg/mL).

### 2.4. Evaluation of Hemolytic Activity and Toxicity Profiles of Antimicrobial Polymers

**Figure 4:**
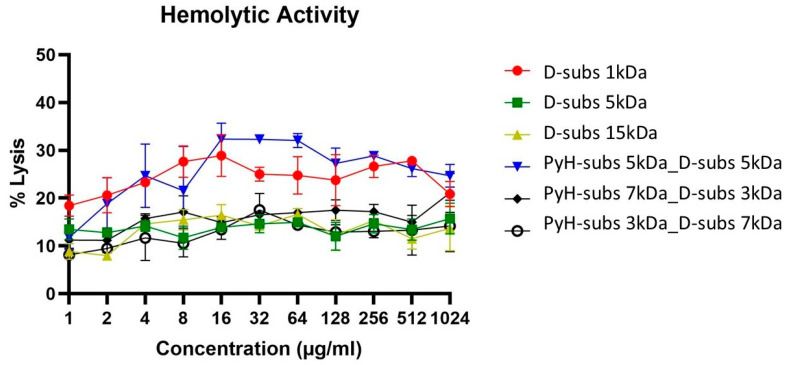
Hemolytic activity of polymers at different polymer concentrations against human erythrocytes. Data are plotted as the mean ± SD of three replicates.

The toxicity profiles of the antimicrobial polymers were evaluated using two complementary assays: the MTT assay to assess cytotoxicity and a hemolysis assay to measure blood cell lysis to determine their hemolytic potential. Hemolytic activity was quantified as the percentage of red blood cell lysis and is presented in Figure 4 based on polymer concentrations ranging from 1 to 1024 µg/mL. In this figure, the *y*-axis indicates 50% hemolysis, serving as a reference point for HC_50_ values. Notably, none of the polymers reached or exceeded the HC_50_ threshold at the maximum tested concentration of 1024 µg/mL, indicating relatively low hemolytic activity.

**Figure 5:**
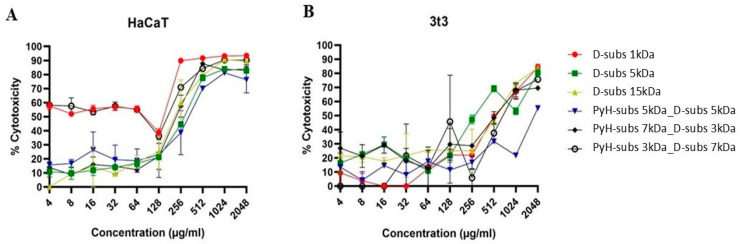
Dose-dependent cytotoxicity profiles of antimicrobial polymers against (**A**) HaCaT cell line and (**B**) 3t3 cell line. Cytotoxicity data are plotted as the mean ± SD of three replicates.

HaCaT and 3T3 cell lines were treated with increasing concentrations of the synthesized polymers, ranging from 4 to 2048 µg/mL, for 24 h (Figure 5A,B). As shown in Figure 5A, the D-subs 1kDa and PyH-subs 3kDa_D-subs 7kDa polymers exhibited comparable cytotoxicity profiles, whereas the remaining polymers demonstrated a dose-dependent increase in cytotoxic effects on the HaCaT cells. The half-maximal inhibitory concentration (IC_50_) values were calculated for all polymers in both cell lines. Notably, the polymers displayed lower cytotoxicity toward 3T3 cells compared with HaCaT cells. Despite this, no statistically significant differences were observed between the IC_50_ and MIC values for the polymers, with the exception of their activity against *S. aureus*. The selectivity index (SI), calculated as the ratio of IC_50_ to MIC for *S. aureus*, is presented in Table 4. Among the tested polymers, PyH-subs 5kDa_Dsubs 5kDa demonstrated the highest selectivity index, with a value of 222.5.

### 2.5. Stability Profiles of Polymers

The stability of antimicrobial polymers in physiologically relevant media is critical for their potential biomedical applications. To evaluate their environmental robustness, a representative example, the low-molecular-weight DABCO homopolymer with a low molecular weight, D-subs 1kDa, was incubated in phosphate-buffered saline (PBS, pH 7.4) and physiological saline solution (SF) media at 37 °C for predetermined time intervals. Conducting stability studies of polymers in PBS and SF media at various temperatures over extended time periods using HPLC is crucial for evaluating their physicochemical integrity and degradation behavior under physiologically relevant conditions. These experiments help to establish whether the polymer retains its structural and functional properties in environments that mimic in vivo conditions. Temperature-dependent stability assessments provide insight into the material’s suitability for storage, transportation, and eventual clinical application.

The stability of D-subs 1kDa in PBS was analyzed at 4 °C, 37 °C, and room temperature conditions for the 7th, 15th, and 28th days.

**Figure 6:**
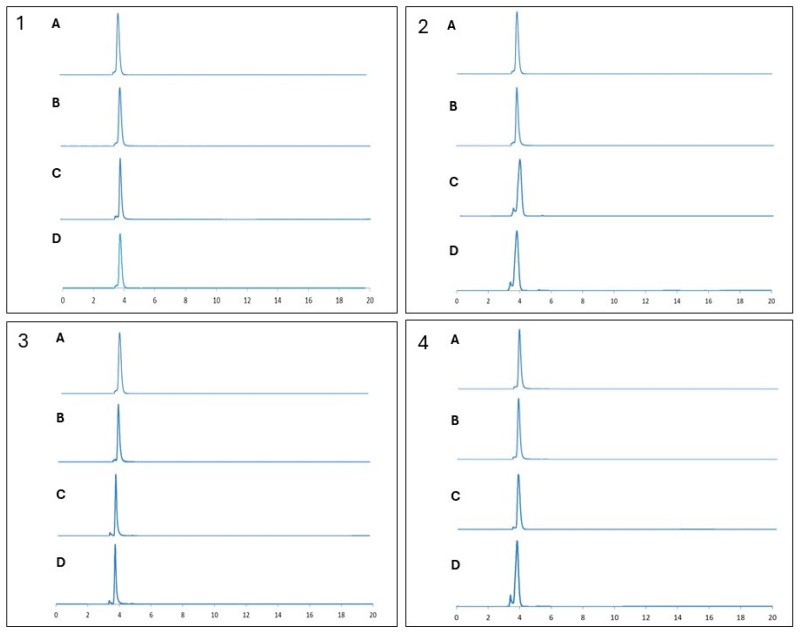
HPLC chromatograms presenting stability profiles of D-subs 1kDa in (**1**) PBS at +4 °C, (**2**) PBS at +37 °C, (**3**) PBS at RT °C, and (**4**) SF at +37 °C on (A) Day 0, (B) Day 7, (C) Day 15, and (D) Day 28.

For all conditions, polymers were analyzed with RP-HPLC and the first day samples were used as a reference. According to the RP-HPLC results, the retention times of polymers in PBS stay stable for at least 28 days at different temperatures (Figure 6(1–3)). In Figure 6(4), the RP-HPLC results showed that the polymers in SF remained stable for at least 28 days at 37 °C in PBS, as well.

**Figure 7:**
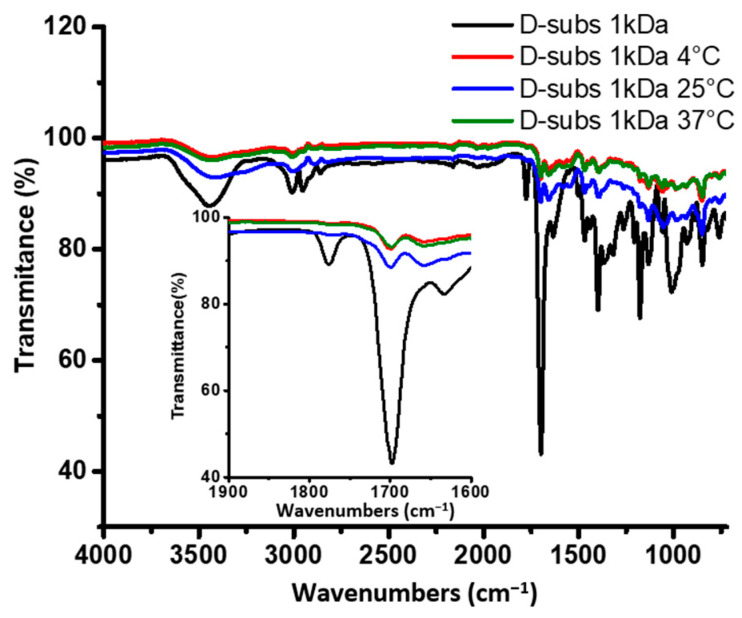
Overlapping FTIR spectra of the D-subs 1kDa homopolymer after stability tests at +4 ± 1 °C, +25 ± 1 °C, and +37 ± 1 °C. Black: D-subs 1kDa (initial); Red: D-subs 1kDa at 4 °C; Navy blue: D-subs kDa1 at 25 °C; Olive: D-subs 1kDa at 37 °C.

**Figure 8:**
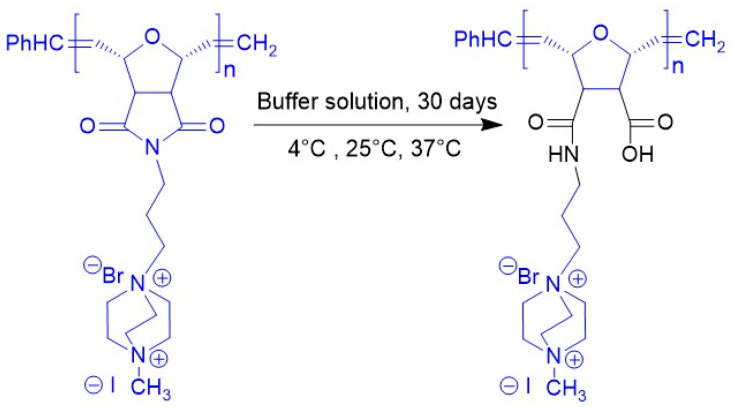
Suggested polymer structure after incubation in buffer for 30 days.

Following one month of incubation in phosphate buffer at three different temperatures, the D-subs 1kDa samples were lyophilized and re-analyzed by FTIR (Figure 7). Comparisons with the original homopolymer revealed that the imide-related peaks at 1318.1 cm^−1^ and 1365.9 cm^−1^ had disappeared. The absorption band corresponding to the C=O stretching of amide (-R–C=O–NH) functional groups at 1697 cm^−1^ also diminished. A possible degradation mechanism of D-subs 1kDa is illustrated in Figure 8.

All polymers exhibited good colloidal and chemical stability in both PBS and SF media, maintaining clear dispersions without visible aggregation or precipitation. No significant changes in color, viscosity, or turbidity were observed during the incubation period. RP-HPLC analysis did not reveal any new signals; however, FTIR analysis confirmed that some structural changes occurred under buffer conditions over a certain period of time.

As part of the stability studies, activity assays were repeated concurrently for polymer samples that had been shown to remain stable for 28 days at different temperatures in SF and PBS media. Biological activity studies conducted with these polymers confirmed that they retained their functionality and did not lose efficacy during the stability testing period.

### 2.6. Morphological Evaluation of S. aureus (SEM)

Scanning electron microscopy (SEM) is a powerful imaging technique that provides high-resolution visualization of surface structures and morphological changes at the micro- and nanoscale. In the context of antimicrobial polymer research, SEM plays a crucial role in elucidating the mechanism of bacterial inactivation by allowing direct observations of structural damage to bacterial cells following treatment [35,40].

**Figure 9:**
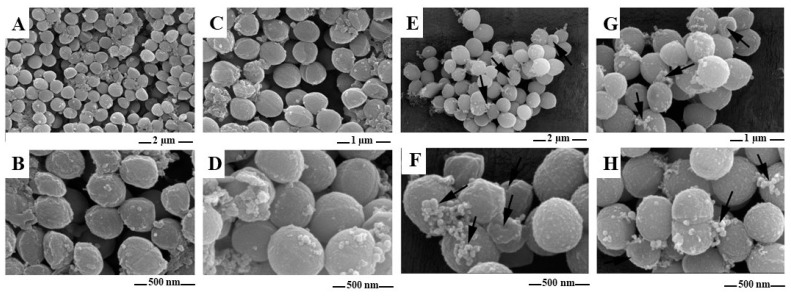
Representative SEM micrographs of experimental groups using *S. aureus*. Control groups of *S. aureus* with a scale of (**A**) 2 µm, (**B**) 1 µm, (**C**) 500 nm, and (**D**) 500 nm. D-subs 1kDa MIC×16 µg/mL applied groups of *S. aureus* with a scale of (**E**) 2 µm, (**F**) 1 µm, (**G**) 500 nm, and (**H**) 500 nm. The arrow indicates the presence of bubble-like protrusions from the cell surface.

In this study, SEM analysis was employed to investigate the morphological alterations in *S. aureus* after exposure to an antimicrobial cationic polymer, D-subs 1kDa. The *S. aureus* cells in the untreated control group exhibited normal morphology, characterized by smooth, circular shapes in SEM micrographs. A topographical analysis of these samples revealed no structural damage to the bacterial surfaces (Figure 9A–D). By contrast, *S. aureus* cells treated with MIC×16 of D-subs 1kDa were compared with untreated controls to assess membrane disruption, cell wall deformation, and leakage of intracellular content—hallmarks of membrane-targeting antimicrobial activity (Figure 9E–H). Although crater-like formations were frequently observed on the bacterial surfaces, no noticeable changes were detected in the overall size or dimensions of the cells. In the *S. aureus* samples treated with MIC×16 of D-subs 1kDa, numerous bacterial cells exhibiting abnormal morphology were observed. Most of these cells displayed vacuole formation within the cytoplasm and evident damage to the cell wall (Figure 9A–D).

### 2.7. Ultrastructural Evaluation of S. aureus (TEM)

TEM is an advanced imaging technique that enables the visualization of internal ultrastructural features of microbial cells at nanometer resolution. Unlike SEM, which provides detailed images of surface morphology, TEM allows for in-depth analysis of intracellular architecture and membrane integrity, making it a valuable tool in antimicrobial research.

In this study, TEM analysis was conducted to investigate ultrastructural changes. TEM imaging was used to compare treated *S. aureus* cells with untreated controls, focusing on alterations such as cytoplasmic leakage, disrupted cell membranes, condensation or disintegration of cytoplasmic material, and compromised cell wall integrity.

**Figure 10:**
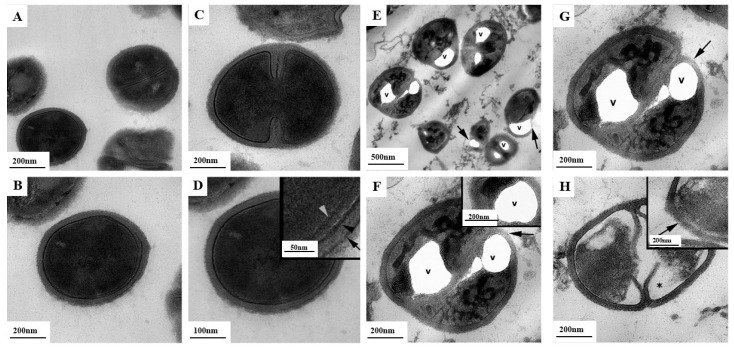
Representative TEM micrographs of experimental groups using *S. aureus*. Control groups of *S. aureus* with a scale of (**A**) 200 nm, (**B**) 200 nm, (**C**) 200 nm, and (**D**) 100 nm. In (**D**), the normal fine structure of the cell wall, cell membrane, and cytoplasm can be seen in the bacterial preparations. The thumbnail shows the cell wall (arrow), outer membrane (black arrowhead), and plasma membrane structure (gray arrowhead) of this group of bacteria in detail. D-subs 1kDa MIC×16 µg/mL applied groups of *S. aureus* with a scale of (**E**) 500 nm, (**F**) 200 nm, (**G**) 200nm, and (**H**) 200 nm. Vacuoles (v) in the cytoplasm, damage to the cell wall structure (arrow), and impaired cytoplasmic content (asterisk) are present in most bacterial cells.

The *S. aureus* cells in the untreated control group exhibited a normal morphology in TEM micrographs (Figure 10A–D). Under low magnification, a few bacterial cells were visible, displaying typical structural features (Figure 10A). The cell wall, membrane structures, and cytoplasm of one *S. aureus* cell appeared intact and well organized (Figure 10B). In Figure 10C, a dividing bacterial cell shows intact morphology with no apparent abnormalities. Overall, the bacterial samples demonstrated well-preserved cell wall, membrane, and cytoplasmic architecture. The inset image in Figure 10D provides a detailed visualization of the cell wall, outer membrane, and plasma membrane of a *S. aureus* from the control group. These structural layers appear continuous and undamaged, indicating healthy bacterial physiology.

After treatment with MIC×16 of D-subs 1kDa, TEM images revealed significant morphological disruptions of the bacteria. Figure 10E shows numerous bacterial cells exhibiting cytoplasmic vacuolization (v), a hallmark of membrane damage and intracellular stress. An arrow indicates disrupted membrane integrity, suggesting the onset of lysis or severe perturbation of the envelope structure. Figure 10F displays enlarged vacuoles (v) and altered cytoplasmic density. The inset confirms the vacuole formation and disintegration of membrane organization, indicating a loss of cellular compartmentalization. Figure 10G highlights severe morphological abnormalities with swollen intracellular vacuoles (v) and membrane rupture (arrow), supporting the idea of a membrane-disruptive mechanism of action by the polymer. Figure 10H further reveals disrupted cytoplasmic content (indicated by an asterisk), suggesting leakage or degradation of cytosolic components. The inset confirms additional cell envelope discontinuity (arrow).

## 3. Discussion

The development of antimicrobial polymers with both high efficacy and low toxicity remains a major challenge in biomedical research. In this study, a design strategy was employed based on the contrasting properties of two monomer units: DABCO-based homopolymers, known for their low toxicity, and hexyl pyridinium-based polymers, which offer potent antimicrobial activity but also exhibit high cytotoxicity. By synthesizing copolymers with varying ratios of these two components, the goal was to achieve an optimal balance between efficacy and safety. This approach aimed at improving the selectivity index (HC_50_/MIC), particularly against *E. coli* and *Candida* species. The experimental results supported this design approach, as PyH-subs 7kDa_Dsubs 3kDa demonstrated strong antimicrobial activity across multiple pathogens while retaining favorable cytotoxicity profiles.

A deviation was observed between the theoretical and experimental molecular weights calculated via end-group analysis. This discrepancy may be attributed to the use of polar coordinating solvents such as DMF and trifluoroethanol during polymerization. These solvents are known to interact with the ruthenium center of Grubbs’ catalysts, potentially leading to sluggish or incomplete initiation and consequently affecting molecular weight control. Despite this discrepancy, the synthesized polymers retained well-defined structures with sufficiently controlled molecular weights, enabling meaningful evaluation of their structure–activity relationships.

Removing residual ruthenium from ROMP-derived polymers is crucial, especially for those intended for biomedical or antimicrobial applications, as Ru is cytotoxic and may hinder regulatory approval for clinical use. Additionally, trace Ru species retained in the polymer matrix can catalyze unintended side reactions, such as cross-metathesis, which may compromise the long-term structural stability of the polymer. The observed molecular weight dependency in Ru removal efficiency suggests that larger polymer chains may facilitate more effective catalyst adsorption or entrapment during purification. In this study, both dialysis and activated carbon treatment were effective in reducing Ru content, with ICP-MS analysis confirming significantly lower levels in samples treated with activated carbon.

Cationic polymers are considered promising antimicrobial agents due to their membrane-targeting, broad-spectrum activity, and low potential for inducing resistance. The differences observed in antimicrobial efficacy among the synthesized ROMP-based cationic polymers can be attributed to structural factors, including the molecular weight and cationic charge density of the polymers. For example, D-subs 15kDa exhibited superior efficacy against both Gram-positive and Gram-negative strains, which was linked to its higher multivalency and ability to form stronger electrostatic interactions with bacterial membranes.

The enhanced activity of D-subs 15kDa may be related to its increased charge density, which can strengthen electrostatic interactions with bacterial membranes, promoting more efficient membrane disruption. The superior performance of PyH-subs 7kDa_D-subs 3kDa suggests that an optimal balance between hydrophobic pyridinium units and hydrophilic/cationic DABCO moieties improves bacterial membrane interaction and penetration. However, excessive hydrophobicity may result in polymer self-aggregation and precipitation in aqueous environments, thereby reducing effective antimicrobial contact.

The intrinsic structural differences between Gram-negative and Gram-positive bacteria also influence susceptibility. Gram-negative strains such as *E. coli* and *P. aeruginosa* possess an additional outer membrane composed of lipopolysaccharides, forming a dual barrier that hinders polymer access. In contrast, *S. aureus*, a Gram-positive bacterium lacking this outer membrane, is more vulnerable to polymer-mediated attacks. This trend was reflected in the MIC results, where lower concentrations of polymers were sufficient to inhibit *S. aureus*, while higher doses were needed for *E. coli* and *P. aeruginosa*.

Toxicity assessments provided further insights into the biocompatibility of the polymers. HaCaT cells, derived from human keratinocytes, and 3T3 cells, derived from mouse embryonic fibroblasts, were selected as representative in vitro models for dermal and general cytotoxicity, respectively [41,42]. All polymers exhibited low hemolytic activity, indicating good compatibility with red blood cells. However, a dose-dependent cytotoxic effect was observed in HaCaT cells, especially for higher molecular weight polymers, emphasizing the need to carefully balance antimicrobial potency with host cell safety. Importantly, IC_50_ and MIC values were generally in close alignment, reinforcing the polymers’ functional selectivity. Among the tested samples, PyH-subs 5kDa_Dsubs 5kDa demonstrated the highest selectivity index (222.5), making it a strong candidate for further biomedical applications.

Polymer stability under physiological conditions is another key consideration for clinical translation. The D-subs 1kDa polymer retained its structural integrity and antimicrobial activity for at least 28 days when stored in PBS and SF at multiple temperatures, confirming its robustness during storage and use. Although RP-HPLC analysis did not reveal degradation products, FTIR spectroscopy suggested partial hydrolysis of labile functional groups, such as imides and amides, over time. These findings highlight FTIR’s sensitivity in detecting subtle structural changes that may not be visible through conventional chromatographic techniques. Nevertheless, bioactivity assays performed after a 28-day incubation confirmed that the antimicrobial properties of the polymers remained intact, indicating that any chemical alterations had limited functional impact.

Finally, morphological and ultrastructural evaluations supported the proposed mechanism of action of the D-subs 1kDa polymer. SEM imaging revealed crater-like deformations, surface roughening, and vacuolization of *S. aureus* cells after D-subs 1kDa polymer treatment—clear signs of membrane damage. Complementary TEM analysis confirmed intracellular disruptions, including vacuole formation, membrane rupture, and cytoplasmic leakage. These observations are consistent with the “carpet model” of action commonly proposed for cationic polymers, wherein electrostatic and hydrophobic interactions lead to membrane destabilization, functional collapse, and cell death [35,40]. The presence of crater-like deformations and membrane collapse in the SEM images of *S. aureus* strongly correlated with MIC-based evidence of polymer potency.

Together, these findings demonstrate that DABCO- and pyridinium-based copolymers synthesized via ROMP offer a promising platform for the development of selective, stable, and biocompatible antimicrobial agents.

## 4. Materials and Methods

### 4.1. Materials

#### 4.1.1. Instruments

FT-IR spectra were recorded using an ATR accessory on a Perkin–Elmer (Waltham, MA, USA) FT-IR spectrophotometer in the wavenumber range of 4000–650 cm^−1^.

^1^H NMR spectra were obtained using a Bruker (Billerica, MA, USA) 500 MHz NMR spectrometer.

The zeta potentials of the polymers were measured using a Malvern (Worcestershire, UK) Zetasizer Nano ZS (633 nm laser, 175° scattering angle, −172.2 toluene count rate).

Ruthenium (Ru) content was determined using an inductively coupled plasma optical emission spectrometer (ICP-OES) model Shimadzu (Kyoto, Japan) ICPE 9000.

#### 4.1.2. Monomer and Polymer Synthesis

Chemicals used in the experiments were purchased from Sigma-Aldrich (St. Louis, MO, USA) and used without further purification unless otherwise stated.

Furan, tetrahydrofuran, diethyl ether, chloroform, ethyl acetate, hexane, petroleum ether, methanol, methyl iodide, acetonitrile, pyridine, bromo hexane pentane, N,N-dimethylformamide, dichloromethane, ethyl vinyl ether, trifluoroethanol, and dimethyl sulfoxide were purchased from Sigma-Aldrich (St. Louis, MO, USA). Maleic anhydride, 3-bromopropylamine hydrobromide, sodium sulfate, sodium chloride, sodium bicarbonate, DABCO, Grubbs 2nd generation catalyst, 3-bromopyridine, and silica gel were also obtained from Sigma-Aldrich (St. Louis, MO, USA). Hexane and ethyl acetate were distilled prior to use.

Grubbs 3rd catalyst was freshly synthesized using Grubbs 2nd generation catalyst [43].

#### 4.1.3. Bacterial Strains and Cells

*Escherichia coli* (*E. coli*, ATCC 25922) as a fermentative Gram-negative bacillus, *Staphylococcus aureus* (*S. aureus*, ATCC 29213) as a Gram-positive coccus, *Pseudomonas aeruginosa* (*P. aeruginosa*, ATCC 27853) as a non-fermentative Gram-negative bacillus, and *Candida albicans* (*C. albicans*, ATCC 10231) as a yeast fungi were used.

#### 4.1.4. In Vitro Experiments

Mueller–Hinton agar (MHA) (Oxoid, Hampshire, UK), Mueller–Hinton broth (MHB) (Oxoid, Hampshire, UK), ampicillin (GenemarkBio, Taichung City, Taiwan), Dulbecco’s modified Eagle’s medium (DMEM) (Gibco™, Langenselbold, Germany), fetal bovine serum (FBS) (ATCC 30-2020™, Manassas, VA, USA), penicillin–streptomycin (Pen/Strep) (Gibco™, Langenselbold, Germany), magainin-2 (Mag-2) (Sigma-Aldrich, St. Louis, MO, USA), Triton X-100 (Sigma-Aldrich, St. Louis, MO, USA), proteinase k (Qiagen, Hilden, Germany), and the Pierce™ Quantitative Fluoro-metric Peptide Assay (Thermo Scientific™, San Jose, CA, USA) were used.

### 4.2. Methods

#### 4.2.1. Monomer and Polymer Synthesis

The synthesis of monomers. The DABCO-based monomer [35] and the pyridinium-based monomer [34] were synthesized and characterized according to previously reported methods.

The synthesis of polymers. All polymers were synthesized via ROMP using freshly prepared third-generation Grubbs catalyst [43]. For the synthesis of homopolymers (D-subs 1kDa, D-subs 5kDa, and D-subs 15kDa), the reactions were carried out under a nitrogen atmosphere in capped septum vials at room temperature, using dry trifluoroethanol (TFE) as the solvent. The vials were wrapped in aluminum foil to protect the reaction from light. After 24 h of stirring, the polymerization was terminated by adding 0.5 mL of a 1:1 (*v*/*v*) mixture of ethyl vinyl ether (EVE) and methanol, followed by stirring for an additional hour. The resulting polymers were precipitated in diethyl ether, washed via centrifugation, dried under a nitrogen stream, and stored in a desiccator in the dark. The targeted molecular weights for the homopolymers were 1000 g·mol^−1^, 5000 g·mol^−1^, and 15,000 g·mol^−1^, referred to as D-subs 1kDa, D-subs 5kDa, and D-subs 15kDa, respectively.

For the synthesis of the copolymers (PyH-subs 5kDa_D-subs 5kDa, PyH-subs 7kDa_D-subs 3kDa, and PyH-subs 3kDa_D-subs 7kDa), Monomer D and Monomer PyH were dissolved in dry DMF and subjected to the same ROMP procedure as described for the homopolymers. The only difference was the use of ethyl vinyl ether (EVE) alone to terminate the polymerization. The targeted molecular weight for all copolymers was 10,000 g·mol^−1^. The copolymers incorporated double-charged DABCO and hexyl-substituted pyridinium functionalities in feed ratios of 5:5, 3:7, and 7:3, respectively.

#### 4.2.2. Removal of Residual Ruthenium from Polymers

As previously mentioned, the third-generation Grubbs catalyst used for polymerization is a ruthenium-based metal catalyst, which may remain in trace amounts in the final polymer product. Although the literature indicates that ruthenium residues up to 10 ppm are acceptable in pharmaceutical applications [44] efforts were made to further reduce residual ruthenium content following polymer synthesis. Two purification methods were evaluated for this purpose: activated carbon treatment [45] and dialysis using a membrane with a 2000 Da MW cut-off. These methods were tested on two representative polymers, D-subs 10kDa and D-subs 15kDa.

Activated carbon treatment: In a vial, the polymers were dissolved (20 mg/5 mL) in deionized water and 25 mg activated carbon was added to the solution; then, the mixture was allowed to stir in an inert atmosphere for 24 h at room temperature. The next day, a 45-micron filter was attached to the tip of a 5 mL syringe, and the black colored activated carbons were filtered. The resulting water phase was taken into a vial, dried in a vacuum oven at 80 °C for 1 h and 20 min, and weighed for ICP-MS analysis (10 mg).

Dialysis membrane treatment: In a vial, polymers were dissolved (20 mg/3 mL) in deionized water with sonication then 2000 MW cut-off was placed in the dialysis cassette. The dialysis cassette was dialyzed against 250 mL of water in the beaker by changing the water 2 times a day for 2 days. The polymer phase in the cassette was taken into the vial and dried in a vacuum oven at 80 °C for 1 h and 20 min and weighed for ICP-MS analysis (18 mg).

#### 4.2.3. Minimum Inhibitory Concentration of Antimicrobial Polymers

The antibacterial efficacy of the tested bacterial strains was assessed using the broth microdilution technique in accordance with the by European Committee on Antimicrobial Susceptibility Testing (EUCAST) [46]. Bacterial strains were cultured on Mueller–Hinton Agar (MHA) and incubated overnight at 37 °C. Stock polymer solutions were adjusted at 2048 µg/mL and subjected to two-fold serial dilutions down to 0.125 µg/mL. Each experiment was performed in triplicate to ensure reproducibility.

Antifungal activity against Candida species was evaluated following the standardized protocol outlined in the CLSI M27-A3 guideline [47]. DABCO-based homopolymers were dissolved in water and subsequently diluted in buffer. For the copolymer series containing both DABCO and PyH units, a minimal amount of DMSO was used to facilitate dissolution, after which the polymers were added to the buffer solution. The potential effects of DMSO concentration were also evaluated and included as a control in both the antibacterial and hemolysis assays.

#### 4.2.4. Hemolytic Activity of Antimicrobial Polymers

Approval to use fresh human blood in this study was obtained from the Acibadem Mehmet Ali Aydinlar University Local Ethics Committee (Approval No. 2023-2/32, dated 27 January 2023). Initially, 30 µL of freshly collected male human blood was diluted in 10 mL of autoclaved TBS (Tris-buffered saline; 10 mM Tris, 150 mM NaCl, pH 7.2) and centrifuged three times at 1500 rpm for 5 min. Polymer solutions, ranging in concentration from 1 to 1024 µg/mL (100 µL total volume) were serially diluted, mixed with 100 µL of the prepared blood suspension, and incubated at 37 °C for 30 min. Each polymer concentration was tested in triplicate. Following incubation, the 96-well plate was centrifuged at 1500 rpm for 10 min. Hemolytic activity was assessed by measuring the absorbance at 414 nm using a microplate reader (Gen5 Synergy HT, BioTek, Winooski, VT, USA) [36,42].

The percentage of hemolysis induced by each peptide concentration was calculated using the following formula:

% lysis = OD_414_ − OD_414_ (blank)/OD_414_ (total lysis − blank) × 100

#### 4.2.5. Toxicity Profiles of Antimicrobial Polymers

The 3T3 (CRL-1658™, ATCC^®^, Manassas, VA, USA) and HaCaT (PCS-200-011™, ATCC^®^, Manassas, VA, USA) cell lines were cultured in DMEM supplemented with 10% FBS and 1% Pen/Strep. Each well of a 96-well plate was seeded with 5 × 10^4^ cells and incubated at 37 °C using 5% CO_2_. The polymer samples were tested in triplicate across a concentration range of 0.5 to 32 µg/mL, with incubation maintained for 24 h. Untreated cells and Mag-2 peptide were used as negative and positive controls, respectively. Following the incubation period, cytotoxicity was assessed using the MTT cell proliferation assay kit (Roche, Basel, Switzerland). Absorbance measurements were taken at 550 nm with background correction at 690 nm using a microplate reader (Gen5 Synergy HT, BioTek) [42,48]. The half-maximal inhibitory concentration (IC_50_) was calculated as the peptide concentration required to reduce cell viability by 50%.

#### 4.2.6. Stability Profiles of Antimicrobial Polymers

Stability experiments were conducted using the D-subs 1kDa polymer. The polymer was prepared at a concentration of 2 mg/mL and mixed with PBS or SF with a 1:9 ratio. PBS-based stability tests were carried out at RT, 4 °C, and 37 °C, while SF-based tests were conducted at 37 °C.

For enzymatic degradation studies, the concentration of D-subs 1kDa polymer was prepared 1 mg/mL in TBS buffer (pH 7.6). Polymer to enzyme mixtures were prepared at a ratio of 1:10 and incubated at 37 °C with shaking at 150 rpm overnight. At the end of the incubation periods, the samples were analyzed using RP-HPLC with an AdvanceBio Peptide Plus column (675950-902, Agilent Technologies, Santa Clara, CA, USA) [49]. The following mobile phase was used for isocratic elution at 236 nm: 25 mM K_2_HPO_4_: Acetonitrile (91:9; *v*:*v*).

To assess the biological activity of the polymers following protease treatment, minimum inhibitory concentration (MIC) assays were also performed.

FTIR analyses related to the stability studies of the D-subs 1kDa homopolymer were performed as follows: D-subs 1kDa polymer was dissolved in phosphate-buffered saline (PBS, pH 7.4) to a final concentration of 5 mg/mL. The polymer solutions were stored at three different temperatures: 4 ± 1 °C, 25 ± 1 °C, and 37 ± 1 °C for a period of 30 days under controlled relative humidity conditions in a refrigerator and a stability chamber. At the end of the storage period, the polymer samples were lyophilized, and the dried polymer samples were recharacterized using FTIR spectroscopy.

#### 4.2.7. Scanning Electron Microscopy (SEM) and Transmission Electron Microscopy (TEM) Analysis

SEM analysis was performed using a dialysis membrane to entrap *S. aureus* (ATCC 29213). Subsequent procedures were carried out in accordance with the methodology described by Kocagoz et al. [42]. Samples were visualized using a Thermo Scientific Quattro SEM at scales of 2 µm, 1 µm, 500 nm, and 500 nm [50].

For TEM analysis, S. aureus (ATCC 29213) samples were fixed using a 2.5% glutaraldehyde solution prepared in 0.1 M PBS at pH 7.2. Subsequent procedures were carried out in accordance with the methodology described by Kocagoz et al. [42]. Analyses were performed using Thermo Scientific TEM (Talos L120C) with scales of 200 nm and 500 nm [51].

## 5. Conclusions

This study demonstrates the successful synthesis and characterization of DABCO-based cationic homo- and copolymers with significant antimicrobial efficacy and favorable biocompatibility profiles. In particular, the DABCO homopolymer, D-subs 15kDa, and hexyl pyridine PyH-substituted copolymer variants, exhibited potent activity against both Gram-positive and Gram-negative bacteria, while maintaining low hemolytic and cytotoxic effects. Their primarily mechanism of action, membrane disruption, was confirmed via SEM and TEM imaging, consistent with the hypothesized “carpet model” of action common to cationic antimicrobial agents. Notably, these polymers maintained their structural integrity and antimicrobial performance under physiologically relevant conditions, highlighting their potential for long-term clinical application. Given their potent antimicrobial activity and low cytotoxicity, these DABCO-based cationic polymers are being further developed into a topical cream formulation. Preclinical studies using a mouse model of skin infection are currently underway to evaluate their in vivo efficacy.

## Figures and Tables

**Table 1:** Summary of theoretical M_n_ calculated M_n,_ and zeta-sizer values of the polymers.

Polymer	*m*/*n* NMR	M_n_, Theoretical [a]	M_n_, NMR [b]	d.nm, Zeta-Sizer [c]
D-subs 1kDa	-	1000	1239	340.3
D-subs 5kDa	-	5000	3304	494
D-subs 15kDa	-	15,000	7434	1158
PyH-subs 5kDa_D-subs 5kDa	0.69	10,000	6574	1076
PyH-subs 7kDa_D-subs 3kDa	0.82	10,000	8548	173.5
PyH-subs 3kDa_D-subs 7kDa	0.88	10,000	13,167	1199

*m*: number of repeating units for monomer D. *n*: number of repeating units for monomer PyH. *m*/*n*: molar feed ratio of copolymers calculated from ^1^H NMR. [a] Theoretical average molecular weight of the polymer (g mol^−1^). [b] M_n_, NMR: The average molecular weight of the polymers calculated from ^1^H NMR (g mol^−1^). [c] Particle size (d.nm) determined using zeta-sizer.

**Table 2:** Residual Ruthenium metal % after removal treatments.

Polymer	Decreasing Ruthenium (%)	
	Dialysis Membrane	Active Carbon
D-subs 10kDa	34.3 ± 3.3%	63.6 ± 1.9%
D-subs 15kDa	66.9 ± 4.1%	83.2 ± 0.9%

**Table 3:** MIC (µg/mL) values of antimicrobial polymers against *E. coli* ATCC 25922, *S. aureus* ATCC 29213, *P. aeruginosa* ATCC 27853, and *C. albicans* ATCC 10231.

Polymer	*E. coli*	*S. aureus*	*P. aeruginosa*	*C. albicans*
D-subs 1kDa	256	8	256	512
D-subs 5kDa	128	8	128	512
D-subs 15kDa	16	16	64	256
PyH-subs 3kDa_D-subs 7kDa	256	16	1024	516
PyH-subs 5kDa_D-subs 5kDa	128	8	256	1024
PyH-subs 7kDa_D-subs 3kDa	32	16	32	256

**Table 4:** IC50 and selectivity index of the polymers. The IC50 data were calculated as an average of three replicates.

Polymer	HaCaT IC_50_ (µg/mL)	*S. aureus* SI *	3t3 IC_50_ (µg/mL)	*S. aureus* SI *
D-subs 1kDa	234.82	29.35	646.29	80.78
D-subs 5kDa	267.86	33.48	244.25	30.53
D-subs 15kDa	214.26	26.78	612.95	76.61
PyH-subs 5kDa_D-subs 5kDa	264.29	33.03	1780	222.5
PyH-subs 7kDa_D-subs 3kDa	218.37	27.29	483.29	60.41
PyH-subs 3kDa_D-subs 7kDa	253.84	31.73	870	108.75

* SI is the selectivity index calculated as the IC_50_/MIC value of *S. aureus*.

## Data Availability

The original contributions presented in this study are included in the article. Further inquiries can be directed at the corresponding authors.

## Abbreviations

The following abbreviations are used in this manuscript:

MIC	Minimal Inhibitory Concentration
SEM	Scanning Electron Microscopy
TEM	Transmission Electron Microscopy
DMF	Dimethylformamide
MHA	Mueller–Hinton Agar
